# Mendelian Randomization and Type 2 Diabetes

**DOI:** 10.1007/s10557-016-6638-5

**Published:** 2016-01-26

**Authors:** Daniel I. Swerdlow

**Affiliations:** Department of Medicine, Imperial College London, London, UK

**Keywords:** Type 2 diabetes, Mendelian randomisation, Risk factors, Causal inference

## Abstract

Type 2 diabetes (T2DM) is a common, complex disease that poses a substantial burden on individual and population health, but we have relatively limited understanding of its underlying pathophysiology. Observational studies have highlighted large numbers of risk factors for T2DM, some of which are modifiable through behavioural or pharmacological intervention. Determining which of these risk factors plays a causal role in the development of T2DM has been a challenge, but Mendelian randomisation (MR) studies are harnessing genetic data in population studies to offer new insights. Using evolving analytical methods, MR studies continue to address questions of causality related to T2DM, including exploring the roles of adiposity, blood lipids and inflammation. The causal roles of a number of important modifiable risk factors have been confirmed by MR studies, while the relevance of others has been called into question. As more MR studies are conducted, methods are developed and refined in order to make the most efficient and reliable use of available genetic and phenotypic data. In this review, the design and findings of some important MR studies related to T2DM are explored and their relevance for translation to clinical practice considered.

## Type 2 Diabetes – a Complex Disease

Observational epidemiology over several decades has provided a broad view of the risk factors for type 2 diabetes mellitus (T2DM). For example, it is clear that advancing age, greater body mass index (BMI) [1], certain dietary habits [2] and lower physical activity [3] are associated with higher T2DM risk. In more recent years, genome-wide association (GWA) studies have offered novel insights into the genetic architecture underlying the pathophysiology of T2DM. These studies have confirmed that, as a so-called ‘complex disease’, the development of T2DM appears to be influenced by a wide range of biochemical, genetic, behavioural and environmental determinants, each of which individually contributes only a portion of disease risk [4]. Importantly, however, observational studies have largely been unable to attribute causality robustly to a given risk factor. This picture of multi-factorial aetiology is similar to that found in other complex diseases such as coronary heart disease (CHD) or stroke. Causal roles for a number of important risk factors in cardiovascular disease (CVD), such as higher LDL cholesterol (LDL-C) or blood pressure, have been demonstrated through strong evidence from randomised controlled prevention trials [5, 6]. In contrast, despite the wealth of treatment trials of hypoglycaemic interventions for individuals with T2DM, comparable findings from prevention trials have not been reported in T2DM. As a consequence, reliable, population-based evidence confirming the underlying causes of T2DM has, until recently, been relatively scarce. Using common genetic variants, often those identified by large GWA studies, Mendelian randomisation (MR) studies have begun to offer new evidence for causal mediators in the development of T2DM. In this review, we consider the role MR has to play in investigating a complex disease such as T2DM, and important findings from recent MR studies in this area.

## Introduction to Mendelian Randomisation

As noted above, observational epidemiological studies have a valuable role in identifying risk factors associated with T2DM. By virtue of their design, however, observational studies are unable to offer reliable evidence for causal relationships between observed risk factors and a given disease outcome [7]. This limitation results from the propensity of observational studies for bias (in a number of forms), confounding (both measured and unmeasured), and reverse causation (whereby an observed risk factor is, in fact, a consequence of the disease process rather than a contributor). The classical randomised controlled trial (RCT) design overcomes these three hurdles through random allocation of participants to treatment or control groups (thus avoiding confounding), blinding of investigators and/or participants to trial group allocation (avoiding a number of influential biases), and through their inherently prospective nature (which overcomes the possibility of reverse causation).

MR studies exploit inherent properties of common genetic variation to estimate the causal contribution of a risk factor to risk of a given disease outcome [8–10]. Crucially, MR relies on the random allocation of alleles at the time of conception, and the independent assortment of parental alleles, as described by Gregor Mendel [11]. These near-universal features of genetic variation help to avoid confounding in MR studies in the same way as random treatment allocation does in RCTs. Furthermore, individuals are largely unaware of their genotype at a given locus, which helps to overcome several biases, and the flow of biological influence in general follows a unidirectional path from genome sequence through transcription, translation and downstream to complex phenotypes, thus generally avoiding reverse causation.

The MR model examines three relationships:

i. the observed association between the exposure and the outcome;
ii. the association between a genetic variant (or a group of variants) and the exposure; and,
iii. the association between the genetic variant(s) and the outcome.

The model rests on the assumption that the genetic variant (termed the ‘instrument’) associates exclusively with the exposure and not with potential confounders. If all three of the relationships above can be demonstrated in an appropriately sized sample, a role for the exposure in causing the outcome can be inferred by formally synthesising a ‘causal estimate’. The genetic instrument is usually either a single nucleotide polymorphism (SNP) or a group of SNPs combined to form a composite instrument, or score [12, 13]. The genetic instrument should, in general terms, have a sufficiently large influence on the exposure of interest to allow its effect to be detected in the available sample and have minimal effects on other variables that could confound the exposure-outcome relationship. In the examples of MR studies discussed below, a variety of approaches to instrument formulation are used, each with important advantages and limitations.

## Endogenous Risk Factors for T2DM

### Obesity and Adiposity

The majority of MR studies in T2DM have focussed on determining whether traditional risk factors have a causal role in the disease aetiology or are merely bystanders. Among the risk factors attracting most attention is adiposity, since higher BMI is a well-described risk factor for T2DM [1], and a number of large-scale MR studies have addressed this relationship. BMI is a complex phenotype with many determinants, including variants several at genetic loci [14]. On the genetic level at least, this is in stark contrast with comparatively simpler phenotypes such as C-reactive protein (CRP), a circulating protein that is the product of a single gene [15]. Some MR studies investigating the role of BMI on disease risk have used as instruments a limited number of variants in the genes most strongly associated with BMI, such as *FTO* and *MC4R*. A more comprehensive and robust approach has developed more recently, whereby a larger set of BMI-associated variants are combined into an ‘allele score’ or ‘genetic risk score’ and used as the genetic instrument in the MR analysis. The score-based method has the advantage of increasing the effect size of the genetic instrument on the exposure, and may also reduce bias [12, 13].

An early analysis of this type used a single variant at the *FTO* locus with a strong and well-characterised association with higher BMI [16], each allele accounting for approximately 0.29 kg/m^2^ higher BMI in a large GWA study (*p* = 4.40 × 10^−7^; *n* = 127,553) [17] and 0.36 kg/m^2^ in the MR study (*p* = 4.3 × 10^−52^; *n* = 198,502). Using this strong genetic instrument, the MR study reported a causal role for higher BMI in combined incident and prevalent T2DM risk, with an odds ratio (OR) of 1.37 (95 % confidence interval, CI, 1.23 to 1.51; *p* = 2.0 × 10^−9^; 20,804 cases, 139,543 controls) per 1 kg/m^2^ increase in BMI. Furthermore, the authors reported a similar role for BMI in the risk of metabolic syndrome (OR 1.31; 95 % CI 1.18 to 1.45; *p* = 2.6 × 10^−7^; 11,608 cases, 37,984 controls).

Two subsequent studies have employed more advanced MR analysis methods and used an allele score as the BMI genetic instrument. The first used a score of 14 BMI-associated SNPs discovered using a cardiovascular gene-centric SNP array [18]; a 1-unit increase in the allele score accounted for a 1.08 kg/m^2^ increase in BMI (95 % CI 0.95 to 1.21; *n* = 34,538) [19]. Using this score, a 1 kg/m^2^ genetically instrumented increase in BMI was associated causally with higher T2DM risk (OR 1.27; 95 % CI 1.18 to 1.36; 4407 cases, 31,844 controls). In the second major MR study, the authors sourced the SNPs for their allele score from a large GWA study of BMI [20], and included 32 variants [21]. In a meta-analysis sample of 81,764 individuals from 25 studies, each additional allele in the score led to a standardised 0.030 SD increase in BMI (95 % CI 0.028 to 0.030; *p* = 2.8 × 10^−109^). Although the authors did not examine associations of the score with risk of T2DM itself, they demonstrated causal associations of higher BMI with higher fasting plasma glucose, higher post-oral glucose tolerance test (OGTT) plasma glucose, higher haemoglobin A_1c_ (HbA_1c_), and higher fasting insulin, suggesting a strong relationship of BMI with a dysglycaemic phenotype. These three MR studies have employed a range of analytical techniques to demonstrate a consistent causal role for higher BMI in increasing T2DM risk, which is in keeping with the longstanding findings from both traditional observational epidemiology and the clear relationship of greater adiposity with insulin resistance.

### Systemic Inflammation

A role for inflammation in the development of T2DM has been proposed for many years on account of the observed relationships between higher concentrations of biomarkers of inflammation, such as CRP and interleukin-6 (IL-6), and T2DM risk [22]. As suggested above, this relationship may result from confounding, or from reverse causation; for example, higher BMI is known to be associated both with systemic inflammation [23], and via a causal pathway with T2DM. MR studies have sought to investigate this, focussing on three important biomarkers of inflammation: CRP, IL-6 and interleukin-1 (IL-1).

An early MR study in a sample of 3218 women used haplotypes in the *CRP* gene as instruments to investigate the role of CRP in the metabolic syndrome [24]. The study reported causal associations of a doubling of CRP concentration with lower BMI (−0.44 kg/m^2^; 95 % CI –1.34 to 0.46), and with higher HOMA-IR, a measure of insulin resistance (0.94; 95 % CI 0.84 to 1.07). There was, however, no causal association with other components of the metabolic syndrome, including systolic blood pressure, waist:hip ratio, HDL-C and triglycerides. The authors concluded that these conflicting findings did not support a causal role for CRP per se in the development of the metabolic syndrome, despite strong observational evidence linking the two. A subsequent, larger study again used SNPs in the *CRP* gene as instrumental variables and found no genetic associations with HbA_1c_, HOMA-IR, or risk of T2DM [25]. Although this analysis found that CRP is unlikely to play a causal role in T2DM, the authors suggest that other inflammatory pathways may be aetiologically important. Closely related biologically to CRP is IL-6, a pro-inflammatory cytokine with a large number of physiological effects. The role of IL-6 signalling in cardiovascular disease has attracted widespread attention [26–28], and its influence on dysglycaemia has also been investigated. A large MR study with CHD as its primary endpoint also reported a near-significant effect of a functional variant causing impaired signalling at the IL-6 receptor on lower T2DM risk [26]. In a large GWA meta-analysis, however, the same functional variant was found not to be associated with T2DM risk (OR 1.03; 95 % CI 0.99 to 1.05; *p* = 0.18; 9580 cases, 53,810 controls; data available from http://diagram-consortium.org/downloads.html) [29]. IL-1 signalling lies upstream of IL-6-mediated pathways and has also been investigated for a potential role in T2DM aetiology. Of note, canakinumab, a monoclonal antibody inhibiting IL-1β, has been shown in a small RCT (*n* = 67) to have no effect on improving glycaemic control in recently diagnosed type 1 diabetes [30]. A large MR meta-analysis, used variants in the *IL1RN* gene, which encodes IL-1 receptor antagonist (IL-1Ra), the naturally occurring inhibitor of the IL-1 receptor [31]. Although the genetic instruments were strongly associated with IL-1Ra concentration, there was no association with T2DM risk when the variants were combined into a score (OR 0.99; 95 % CI 0.97 to 1.01; *p* = 0.47; 18,715 cases, 61,692 controls). Although the inflammatory hypothesis in T2DM aetiology appears plausible, evidence from MR studies has so far failed to support it. It is possible, nonetheless, that larger studies and investigation of other inflammatory pathways may yield different findings.

### Blood Lipids

The relationship of blood lipids with T2DM risk has risen in prominence in recent years, catalysed largely by the finding that statin therapy caused an increase in T2DM risk in cardiovascular disease prevention trials [32, 33]. Furthermore, RCTs of niacin showed higher plasma glucose and T2DM risk [34] and the CETP inhibitor torcetrapib showed a beneficial effect on glycaemic control in the ILLUMINATE trial [35]. The link between lipid metabolism and glycaemic control appears to be strengthening, and MR studies are shedding some light on its underlying mechanisms [36].

An observed relationship between higher circulating triglyceride concentration and higher T2DM risk has been recognised for several years [37], although whether this reflected a causal association was unclear. MR studies of blood lipids pose similar methodological challenges to those investigating BMI since blood lipid fractions are influenced by variants at a large number of genetic loci [38]. Studies have used different approaches to overcome these challenges, some of which are illustrated in the studies described here. A consortium-based MR study including data from four cohorts demonstrated that despite a strong observational association between circulating triglyceride concentration and T2DM risk, fasting insulin and glucose and HOMA-IR, there was no association with a robust triglyceride allele score instrument incorporating information from 10 variants across 9 loci [39]. In a Pakistani sample of 2131 individuals, a similar observed relationship was found between triglycerides and T2DM risk, however an allele score of 10 SNPs showed no association with T2DM risk (OR 0.97; 95 % CI 0.91 to 1.04; *p* = 0.41) [40].

The relevance of lipoprotein(a) (Lp(a)) to cardiovascular disease has been demonstrated by MR studies [41], although a specific pharmacological Lp(a) inhibitor has not yet entered advanced clinical development. The role of Lp(a) in T2DM is less clear, but observational studies report an inverse relationship between Lp(a) concentration and T2DM risk. Using a well-characterised variant at the *LPA* locus, the authors of a large MR study reported strong associations of T2DM risk with Lp(a) concentrations, however no evidence of a causal link (OR 1.03; 95 % CI 0.96 to 1.10; *p* = 0.41; 10,088 cases, 68,346 controls) [42]. In the wake of findings from the ILLUMINATE trial of torcetrapib (an agent intended to raise HDL-C to prevent CHD) [35], interest has grown around the relationship of HDL-C with glycaemic control. An MR analysis from Denmark, also using an allele score (nine variants in genes with known roles in HDL-C metabolism) addressed this issue [43]. As expected, the allele score associated strongly with HDL-C, accounting for up to 20 % difference in circulating concentration. There was, however, no causal association seen in the sample of 47,627 individuals, including 2587 patients with T2DM (OR per 0.2 mmol/L reduction in HDL-C 0.93; 95 % CI 0.75 to 1.09).

A larger analysis used publicly-available data from major GWA study meta-analysis consortia [29, 38, 44] to assemble allele scores as instruments for HDL-C, LDL-C and triglycerides [45]. The scores in this analysis were derived from the largest GWA study of lipids conducted to-date (the Global Lipid Genetics Consortium, GLGC) [38], and therefore, arguably, allow the most comprehensive assessment of lipid relationships with T2DM. The analysis showed no causal association between either the HDL-C or triglyceride allele scores of 140 SNPs and T2DM risk, but did reveal a convincing association between the LDL-C SNP score and risk of T2DM. The latter finding of a causal association between higher LDL-C concentration and higher T2DM risk fits well with the emerging strong link between LDL-C modulation and T2DM [36]. As noted above, statin treatment has been shown to increase risk of new-onset T2DM in randomised CVD prevention trials. An MR analysis using common variants in the *HMGCR* gene that encodes HMG-CoA reductase – the intended target of statins – demonstrated that the same variant that associated with lower LDL-C also caused higher T2DM risk, higher plasma insulin and glucose, and higher body weight and BMI [46]. The analysis also compared the genetic effects with those of statin treatment in RCTs on body weight and T2DM and showed a clear directional concordance between the two – both the genetic instruments and statin treatment caused higher body weight and T2DM risk. These findings led to the inference that the effect of statin treatment on T2DM risk was at least partly an on-target effect of the drugs, and was likely mediated via increased adiposity. As the development of novel lipid-modifying drugs, such as the inhibitors of PCSK9, progresses, the possibility of on-target adverse effects on glycaemic control is drawing increasing focus [47].

## Exogenous and Behavioural Risk Factors for T2DM

The MR studies discussed above have all concerned endogenous risk factors – features of human physiology that may influence T2DM aetiology. It is apparent from observational studies that certain behaviours, and particularly dietary preferences, associate with T2D risk. As expected, these exposures are more difficult to address using MR analysis, however a small number of studies have attempted to do so. Common genetic variants have been shown to influence consumption of certain foodstuffs, most notable among these being the association between variants in *ADH1B* (encoding alcohol dehydrogenase 1B) and alcohol consumption [48]. Variants in the gene encoding lactase (*LCT*) have also been associated with differences in consumption of dairy products, and so were used as instruments for milk consumption in an MR analysis that sought to determine whether an observed association between higher dairy consumption and lower T2DM risk was causal [49]. The *LCT* SNP associated weakly with milk consumption, however there was no association of the variant with T2DM risk. The study was impaired by the absence of an internal observed association between milk consumption and T2DM risk, and the modest effect of the genetic instrument, and so should be interpreted cautiously. In a similar vein to the milk consumption MR study, the same Danish group investigated whether coffee drinking played an aetiological role in T2DM [50]. GWA studies have highlighted variants in *CYP1A1*, *CYP1A2* and *AHR* associated with coffee consumption [51] and the MR investigators combined five such SNPs into an allele score as an instrument for coffee drinking. Again, despite a strong observed relationship between higher coffee consumption and lower T2DM risk in a sample of 83,436 individuals, in the MR analysis there was no causal association between the amount of coffee consumed and T2DM risk. The authors conclude that unmeasured confounding likely explained the observed association.

## T2DM as a Risk Factor for Other Diseases

Hitherto we have discussed MR studies that investigated the causal role of a range of risk factors in the development of T2DM. A small number of studies have examined the inverse scenario – the role of T2DM as a risk factor in other diseases, chiefly cardiovascular disease. T2DM is a well-recognised as a potent risk factor for CVD [52], although RCTs of oral hypoglycaemic agents have not demonstrated substantial CVD risk reduction among treated individuals [53]. Two MR studies have included T2DM as the exposure in the putative causal relationship with CHD as the outcome. Using publicly available data from the DIAGRAM [29] and CARDIOGRAMplusC4D [54] consortia to develop and test a 37 SNP allele score for T2DM risk, a 2014 MR study demonstrated a strong causal relationship between higher T2DM risk and higher CHD risk: an increase by 1 in T2DM OR led to an OR for CHD of 1.11 (95 % CI 1.05 to 1.16; *p* = 1.7 × 10^−4^; 63,746 CHD cases and 130,681 controls) [55]. The second analysis adopted a similar approach using data from the GWAS consortia, although the authors used a more permissive strategy in constructing the allele score for T2DM risk, which included 59 SNPs. With the allele score as the independent variable in a regression model, the genetically determined increase in CHD risk resulting from increased T2DM risk was OR 1.63 (95 % CI 1.23 to 2.07; *p* = 0.002). The findings from these MR studies appear to conflict with those from trials of oral hypoglycaemic agents in patients with established T2DM. This may reflect the different consequences of lifelong dysglycaemia on cardiovascular health compared with relatively short-term changes in plasma glucose achieved during a treatment trial. The genetic findings also lend weight to the value of preventing T2DM for reducing risk of CVD later in life.

## Future Prospects for MR in Investigating T2DM

**Fig. 1:**
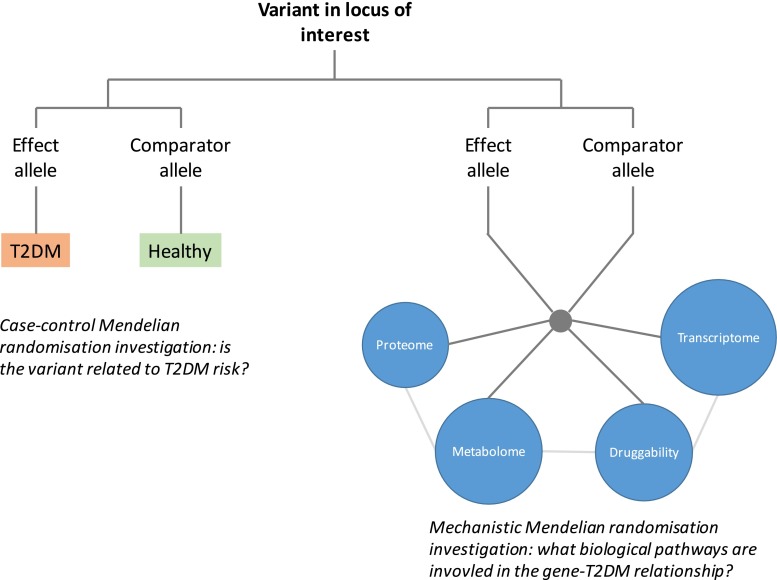
Mendelian randomisation studies in type 2 diabetes. MR studies can help to demonstrate the causal relevance of a biomarker or risk factor to risk of developing T2DM. They can also incorporate proteomic, metabolomics, and transcriptomic data, and other bioinformatics resources in order to investigate the biological pathways linking the gene to disease aetiology

T2DM is an inevitably complex phenotype with many factors contributing to its development. Large numbers of genetic variants are now known to be involved in T2DM risk, and the combined complexity of the genetic architecture and the T2DM phenotype pose important methodological and practical challenges in conducting MR studies. Randomised trials have been able to contribute relatively little to our knowledge of its underlying aetiology but in recent years MR studies have harnessed genetic information to offer new insights. MR studies have confirmed the causal roles of several important, modifiable risk factors such as adiposity in T2DM and so reinforce the utility of preventive interventions for public and individual health. The causal roles of other proposed risk factors have been called into question by MR studies, suggesting these could be downgraded in priority for translation into therapeutic or preventive strategies. GWA studies have identified a number of novel T2DM-associated loci that may represent new therapeutic targets and MR studies offer a valuable opportunity for early validation and prioritisation of these for subsequent development. Furthermore, as metabolomics and proteomic technologies are more widely deployed in population studies and their findings explored together with fine-resolution genetic data, even greater biological insights will be possible (Fig. 1). MR is a growing paradigm with an increasingly credible and reliable role in advancing our understanding of complex disease; T2DM continues to place a substantial burden on health and insights from MR studies are likely to be of increasing importance in our endeavours to understand, prevent and treat it.
